# Choices behind the veil of ignorance in Formosan macaques

**DOI:** 10.1093/pnasnexus/pgac188

**Published:** 2022-09-15

**Authors:** Yi-Ta Lu, Wei-Hsiang Hwang, Yi-Tsung Hsieh, Tsung-Yu Ho, Jian- Da Zhu, Chun-I Yeh, Chen-Ying Huang

**Affiliations:** Department of Economics, National Taiwan University, Taipei 10617, Taiwan; Department of Psychology, National Taiwan University, Taipei 10617, Taiwan; Department of Economics, National Taiwan University, Taipei 10617, Taiwan; Department of Economics, National Taiwan University, Taipei 10617, Taiwan; Department of Economics, National Taiwan University, Taipei 10617, Taiwan; Department of Psychology, National Taiwan University, Taipei 10617, Taiwan; Department of Economics, National Taiwan University, Taipei 10617, Taiwan

## Abstract

An ongoing debate regarding the evolution of morality is whether other species show precursory moral behavior. The veil of ignorance (VOI) paradigm is often used to elicit human moral judgment but has never been tested in other primates. We study the division of resources behind the VOI in Formosan macaques. Monkeys choose the equal division more often when a conspecific is present than when it is absent, suggesting a degree of impartiality. To better understand this impartiality, we measure a monkey’s reactions to two directions of inequity: one regarding inequity to its advantage and the other to its disadvantage. We find that disadvantageous inequity aversion correlates with the degree of impartiality behind the VOI. Therefore, seemingly impartial behavior could result from a primitive negative reaction to being disadvantaged. This suggests a mechanism to explain a tendency toward impartiality.

Significance statement Morality plays an important role in helping people cooperate. To understand the evolutionary basis of morality, we study whether other species show morally relevant behaviors. We run an experiment on monkeys to measure their division of resources. When their own advantage cannot be guaranteed, a stronger aversion to being disadvantaged could make an unequal division unattractive, resulting in an equal division. Therefore, a seemingly moral choice, such as the equal division of resources, could result from an aversion to being disadvantaged. Because an equal division should help cooperation, this suggests an evolutionary account of morality based on cooperation.

## Introduction

According to Darwin (1), morality rests on the need to cooperate. Morality regulates the interactions among individuals and enables smooth cooperation. Smooth cooperation, in turn, benefits individuals. Moral behaviors are hence selected. This evolutionary account raises the interesting question of whether morality is uniquely human, as other species also cooperate. Nonhuman primates are particularly worthy of such investigation due to their evolutionary proximity to humans and sociality.

We study impartial behavior in Formosan macaques (*Macaca cyclopis*). Impartiality is a principle of justice. It focuses on how decisions can be made so that no one is particularly favored. We use a canonical paradigm called the veil of ignorance (VOI) to measure it. This paradigm, modernized by Harsanyi (2, 3) and Rawls (4), is often used to bring about impartial judgments in humans. The key of this paradigm is to elicit judgment in an impersonal way such that as the decision-maker (DM) divides resources, it is ignorant about how much it personally will receive. When a DM divides resources for a conspecific and itself behind the VOI, it only knows that the division applies to them. If the resources are divided unequally, ignorance means one will be advantaged and the other disadvantaged, but the DM cannot know who in particular will be advantaged. Hence, it has no means of dividing resources in a way that directly favors itself. Choices are therefore impersonal. Such impersonal choices, made from behind the VOI, are shorthanded as VOI choices. They reflect the DM’s judgment on what constitutes an impartial division.

To the best of our knowledge, our study is the first VOI experiment on nonhuman primates. Because the VOI paradigm is used to elicit moral judgment in humans, one may wonder whether this paradigm is relevant for animal studies. We cannot elicit a prescriptive moral judgment from animals, but from an evolutionary perspective, the VOI paradigm is relevant. The essence of the VOI paradigm, the ignorance of payoffs, captures an important element when animals make decisions. Because the environment is full of risks, animals may not know how much they will receive when making choices. But their decisions can influence whether they cooperate, which determines their survival. Ignorance of payoffs has been argued to make cooperation possible when ant queens cofound a colony (5) or when a social microbe, *Dictyostelium discoideum*, forms a fruiting body (6). The cooperation increases their survival. Hence, studying VOI choices should better our understanding of biological cooperation and is a worthwhile research topic (7).

In addition to studying VOI choices, we would like to link our research to the literature. Previous research in nonhuman primates focuses on how DM reacts when it is aware of how much it receives compared to a conspecific (8–20). If a DM is other-regarding, perhaps including reacting negatively to inequity, its reaction may lead to a more equitable outcome, which then makes future cooperation more likely. Hence, inequity aversion can regulate cooperation among individuals and is therefore morally relevant. One influential experiment shows that monkeys reject a lesser reward upon observing that a conspecific receives a better one (11). This suggests a specific direction for inequity aversion, called disadvantageous inequity aversion, as the DM receives less than the conspecific. The continuing research debates lively, with some researchers supporting the existence of inequity aversion (11, 14, 19–24) while others do not (13, 15, 16, 18, 25). The literature on this subject is extensive, including the study of several species (8, 10). Moreover, the ways how ecological factors such as kinship (26), social ties (27), and rank (19, 28) influence inequity aversion are examined. Overall, nonhuman primates are inequity averse to various degrees, but no definite conclusion has been reached regarding this subject as a whole.

In light of the VOI concept, previous nonhuman experiments have been conducted in front of the VOI, so to speak, as the DM knows how much it will receive when making the decision. The inequity measured by these experiments is either advantageous when the DM receives more than a conspecific does or disadvantageous when the DM receives less. In contrast, our main experiment is behind the VOI: We elicit impartial judgments when the DM is ignorant of payoffs. Impartiality and inequity aversion are conceptually different. Impartiality aims to achieve justice. The emphasis is to keep choices as fair as possible so that favoritism is unlikely. Inequity aversion, on the other hand, is to see how one reacts by comparing one’s own payoff to that of the other.

The open question we want to address is whether monkeys exhibit impartial choices. Therefore, we start by asking what choice behavior can be considered impartial. Here, two prominent theories and previous nonhuman primate experiments provide useful suggestions. One theory, the maximin principle, recommends prioritizing and hence maximizing the least well-off. This principle is averse to anyone receiving less than the other. A second theory, utilitarianism, does not prioritize anyone. Instead, it advocates maximizing the sum of payoffs for all. These two theories have different implications for resource distributions. The maximin principle suggests an equal division to make the least well-off receive the most. Utilitarianism, on the other hand, is indifferent to any distribution of resources as long as the total sum of resources stays the same. Because moral behavior serves to regulate behavior within a group, the usual practice in the nonhuman primate experiments compares choices of a DM with the presence of a conspecific to those without (14, 15). This motivates the third hypothesis. If we find that a DM divides resources more equally when a conspecific is present than when it is absent, we can take it as evidence of impartiality. Positioning theory-motivated hypotheses along with the hypothesis motivated by the previous experiments helps understand where the choices of the monkeys stand.

Our results support the third hypothesis as monkeys divide resources more equally when a conspecific is present than when it is absent. This leads us to ask the next question of whether choices behind the VOI can be linked to choices in front of the VOI, as the latter has been studied extensively in the literature and better understood. We figure that the ignorance of the VOI choices implies the DM can only know that the division applies to a conspecific and itself. When the resources are divided unequally, one of the two will be advantaged, and the other will be disadvantaged. If the DM is eventually advantaged, then ex post, there is advantageous inequity. On the other hand, if the DM is eventually disadvantaged, disadvantageous inequity arises. This logic suggests that an unequal VOI division could have two possible consequences, depending on whether the DM is eventually advantaged or disadvantaged. Therefore, the VOI choices could be linked to these two directions of inequity. Whether both directions are important for determining VOI choices is an empirical question that our experiment will address.

Overall, we study a suite of choices from both behind and in front of the VOI in Formosan macaques. We hope to contribute to the literature in three respects. Focusing on choices made from behind the VOI, we obtain evidence indicating whether monkeys exhibit impartial behavior. Focusing on choices in front of the VOI, we investigate whether monkeys are inequity concerned, following the literature. Finally, by comparing choices made from behind and in front of the VOI, we draw conclusions on how they are linked. This linkage can extend existing knowledge in two ways. From the perspective of understanding VOI choices, the linkage explains how VOI choices are determined. If we change the perspective to understand inequity aversion, we will show that the linkage suggests the importance of a particular direction of inequity aversion. This importance will lead us to reexamine the mixed results found in the literature.

## Results

The backbone of our experiment was a minidictator game between a DM monkey and a passive recipient monkey (RM). The DM faced two preset choices: one was equal and the other was unequal. It made a choice of distribution of resources for itself and the RM. The RM had no choice. We described the apparatus, the pretests, and the details of the experiment in the “Supplementary Material” section. As a quick summary, in the pretests, we made sure of two things. First, because how the rewards were distributed between the DM and RM was key to this research, we tested whether the DM would take the distribution of rewards into account. Second, we made sure that the DM understood the spinning technique we introduced to implement the VOI condition. We also addressed a prepotent bias observed in primates (15).

We presented the results in four steps. In the first step, we showed that our data were inconsistent with utilitarianism or the maximin principle. Then, by comparing how often an equal division was chosen behind the VOI with a benchmark when the DM was alone, we found that the DM showed signs of impartiality. In the second step, we measured the advantageous and disadvantageous inequity concerns of the DM. In the third step, we linked VOI choices to these inequity concerns. This addressed how VOI choices might be determined and how significant the inequity concerns were. In the fourth step, we speculated on a possible underlying factor for the linkage we observed. We summarized our test results in Table S1 so they could be quickly looked up when necessary.

### Step 1: VOI

In the VOI condition, the DM chose between an equal division and an unequal division. We used grapes as the reward. The DM and RM each received 2 reward units in the equal division. This was represented by (2, 2). The unequal division had 3 reward units for the advantaged and 1 unit for the disadvantaged. We represented this by (3, 1). We counterbalanced the choice display whenever possible.

**Fig. 1.**
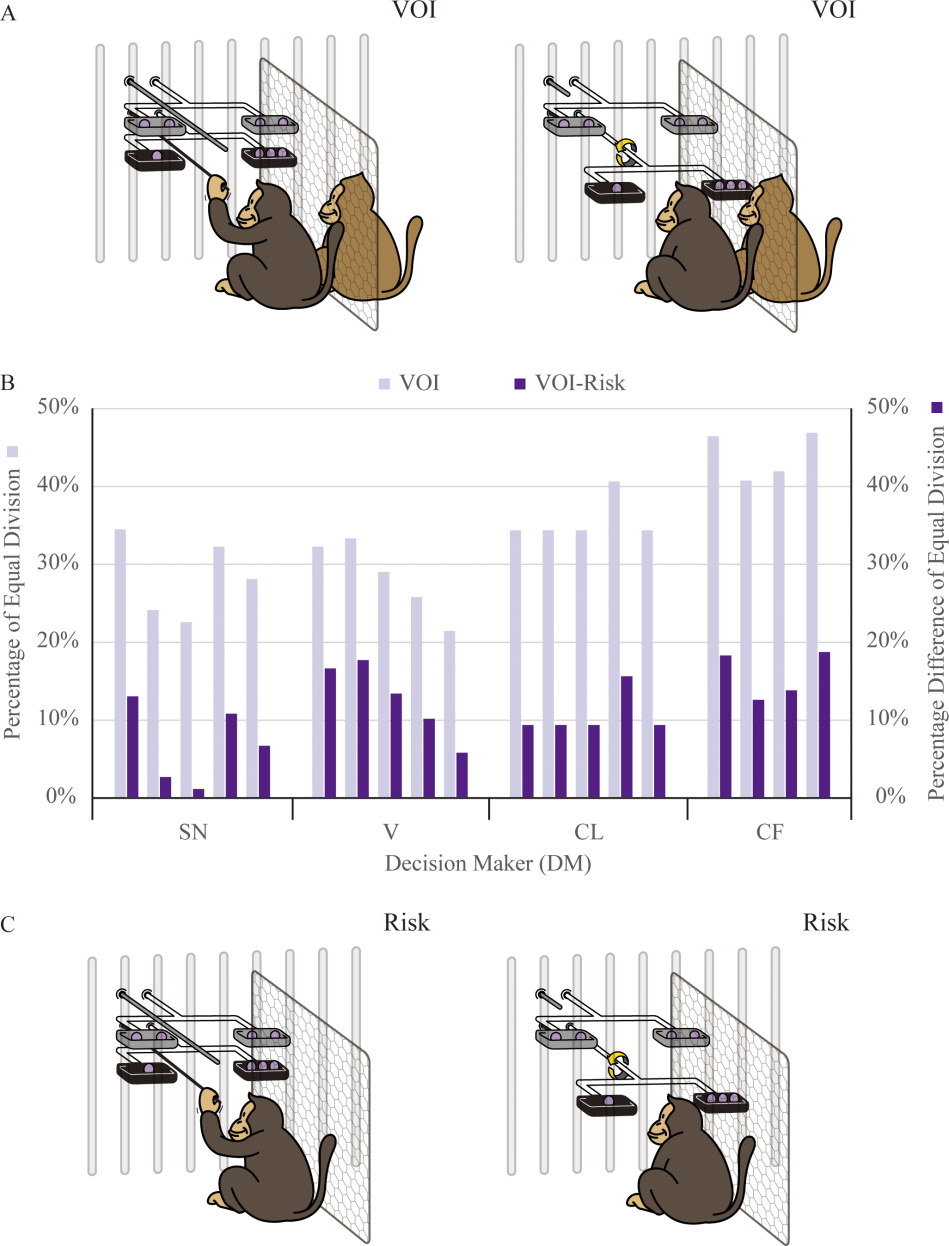
VOI and Risk. (A) Illustration of the VOI condition. The apparatus had upper and lower trays. Each tray contained two food dishes, appearing on the left and the right. We placed in one tray an equally divided reward (2, 2) and in the other an unequally divided one (3, 1). The DM chose between the two divisions. After the choice, half of the time the selected division was spun one loop, so the left dish remained on the left. The other half of the time it was spun one and a half loops, so the right dish came to rest on the left. The DM therefore did not know which dish it would have access to when making a choice. In this illustration, the DM (the dark brown monkey on the left) selected the unequal division, where it would receive 1 or 3 reward units equally likely. If the DM received 1, the RM (the light brown monkey on the right) received 3 and vice versa. We illustrated the case where the bottom tray was baited with the unequal division and 3 were in the right dish. However, we counterbalanced the choice displays. (B) Pair-by-pair breakdowns of the VOI choices and the VOI versus Risk choices. This figure showed the choice percentage for the equal division in VOI (in light purple) and this percentage difference between the VOI and Risk choices (in purple). We grouped pairs of the same DM together and assigned each DM’s name (SN, V, CL, and CF). To facilitate understanding, we ordered the DMs by their body weight so that body weight of the DM increased from left to right. Among the four DMs, monkey SN was lightest, followed by monkey V, monkey CL, and monkey CF. All of the purple bars were positive, indicating that the equal division was chosen more often behind the VOI than in the Risk condition for all pairs. (C) Illustration of the Risk condition. The Risk condition was exactly the same as the VOI condition, except without an RM.

In the VOI condition, the DM should be ignorant of how much it would receive when making a choice. Hence, after the DM made a choice, we spun the selected division. Half of the time, it was spun one loop. The other half of the time, it was spun one and a half loops (Fig. 1A, Movie S1). In Fig. 1A, we illustrated the case where the DM chose the unequal division. After this choice, if the selected division was spun one loop, the DM received 1 unit and the RM 3 units. This would make DM disadvantaged and the RM advantaged. If the selected division was spun one and a half loops, the reverse would occur. The DM received 3 units and was advantaged. In case the equal division was chosen, spinning had no effect, but we still spun for consistency. By spinning the selected division, monkeys could see that a division applied to them, but the DM could be advantaged or disadvantaged. This visually implemented the impersonalization of the VOI and was meant to make it easy to understand (Movie S1). Each DM–RM pair received 32 trials carried out over 4 d.

We ran the VOI condition on 19 pairs of monkeys. This exhausted all possible DM–RM pairs in the lab. On average, DMs chose the equal division (2, 2) 33.56% of the time (SD = 7.41%). Choice frequencies ranged from 21.43% to 46.88% (Fig. 1B). Because both the equal division (2, 2) and the unequal division (3, 1) totaled 4, utilitarianism predicted that the choice frequency of the equal division would be 50%. On the other hand, the least well-off received 2 in the equal division but 1 in the unequal division. Hence, the maximin principle suggested that the choice frequency of the equal division would be 100%. To test whether the choice frequency equaled 50% or 100%, we ran a two-level random intercept regression to estimate the average frequency the equal division was chosen in the VOI condition across DMs. Because any two pairs with the same DM were correlated, the DM was modeled as a random effect to account for it. This was often used for repeated measurements of individuals (29). We explained the details in the “Supplementary Material” section.

The intercept, estimating the average frequency the equal division was chosen in the VOI condition across DMs, was 0.34. This was significantly different from 50% [chi^2^ (*df* = 1) = 24.88, *P* < 0.001] or 100% [chi^2^ (*df* = 1) = 424.35, *P* < 0.001]. Hence, we did not find evidence for these theory-driven hypotheses. We then went on to the third hypothesis, motivated by the usual practice in the previous literature (14, 15). To understand whether this choice frequency was high or low, we compared the VOI condition with a Control condition. The Control condition was exactly the same as the VOI condition, with the single exception that no RM was present (Fig. 1C, Movie S2). As before, a division of (2, 2) gave the DM 2 units no matter how it was spun. Hence, it was a safe choice. A division of (3, 1), on the other hand, gave the DM 3 units half of the time. However, there was no RM for the other 1 unit, the remainder of the division. The other half of the time, the DM received 1 unit. Hence, (3, 1) was a risky choice. The Control condition measured risk preferences and was hence called the Risk condition. Each DM received 32 control trials carried out over 4 d.

Because morality guides behavior to facilitate cooperation with others, the pursuit of self-interest should not be regarded as an indication of morality. In the Risk condition, self-interest was the only possible goal. Hence, if we found no difference between the VOI and Risk conditions, we would conclude that there was no evidence of impartiality. On the other hand, if we found a difference, there were two possibilities. The equal division could be chosen more often in the VOI condition. This case indicated impartiality. On the contrary, if the equal division was chosen less often in the VOI condition, this would suggest the opposite of impartiality or partiality.

#### Impartiality

DMs chose the equal division 22.54% of the time (SD = 5.36%) in the Risk condition. Recall that they chose the equal division 33.56% of the time in the VOI condition. Hence, monkeys chose the equal division more often when an RM was present than when the RM was not present. This was not just true on average. To see that, we measured the degree of impartiality for each DM–RM pair. We took the frequency the equal division was chosen and subtracted the corresponding frequency when the DM was alone in the Risk condition. This frequency difference was taken to reflect the impartiality of a DM in the presence of an RM. In each case, impartiality was positive, as in all 19 pairs, DMs chose the equal division more often in the VOI than in the Risk conditions (Fig. 1B).

To test whether this degree of impartiality was positive, we ran a two-level random intercept regression. The intercept, estimating the average frequency difference between the VOI and Risk conditions across DMs, was positive and significant (intercept = 0.11, *z* score = 7.12, *P* < 0.001, all tests were two-tailed except for the chi-squared tests, *n* = 19 unless otherwise specified). DMs chose the equal division 11% more in the VOI than in the Risk condition, a significant difference. This was a strong indication that the monkeys were influenced by the presence of an RM to choose impartially from behind the VOI.

### Step 2: in front of the VOI

To place impartiality behind the VOI in context, we compared decisions from behind the VOI to those from in front of the VOI. This was done to establish whether it was the resulting inequity that made the unequal division unattractive. From behind the VOI, the choice of an unequal division lead to the DM experiencing advantageous inequity or disadvantageous inequity. On the other hand, if the equal division was chosen, there would be no inequity. It was possible that inequity made the unequal division unattractive and hence the equal division was chosen. With no theory to constrain which direction of inequity would matter for the VOI choices, we studied both types of inequity. If inequity was the reason for impartiality behind the VOI, we would expect to find a link between inequity and VOI choices. This would allow us to speculate on the factor that could explain this linkage.

We ran what we called the Social condition to measure inequity. The setup was similar to that of the VOI, but we did not spin the choice after the DM’s decision was made. Hence, the DM knew how much it would receive and was in front of the VOI.

#### Advantageous inequity

**Fig. 2.**
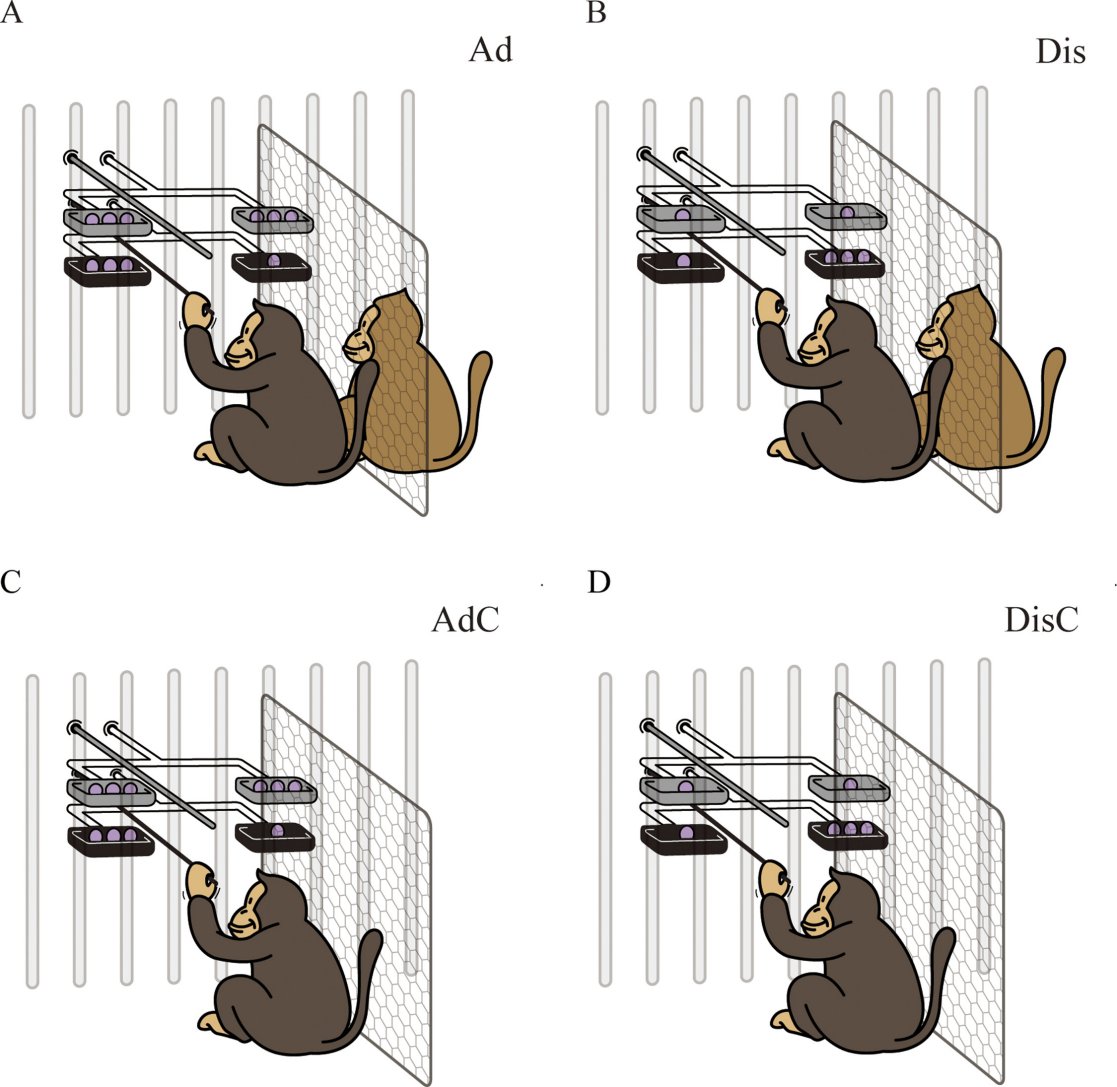
Ad, Dis, AdC, and DisC. (A) Illustration of the Ad condition. The DM chose between the equal allocation (3, 3) and the advantageous allocation (3, 1). Unlike the case of the VOI condition, the selected allocation was not spun; hence, the DM received 3 units, no matter which option it chose. This measured advantageous other-regarding concerns. (B) Illustration of the Dis condition. The DM chose between the equal allocation (1, 1) and the disadvantageous allocation (1, 3). The selected allocation was not spun and the DM received 1 in all cases. This measured disadvantageous other-regarding concerns. (C) Illustration of the AdC condition. It was the control experiment of Ad. It was exactly the same as Ad, except there was no RM. (D) Illustration of the DisC condition. It was the control experiment of Dis and exactly the same as Dis, except there was no RM.

The Social condition had two subconditions: advantageous (Ad) and disadvantageous (Dis). In the Ad subcondition, the DM chose between an equal allocation and an advantageous allocation. The equal allocation, represented by (3, 3), gave the DM and RM 3 units each. The advantageous allocation, represented by (3, 1), gave the DM 3 units and the RM 1 unit. The DM thus had an advantage (Fig. 2A). Because the DM received 3 units in both allocations, self-interest had no role. If the DM was averse to being advantaged over the RM, it would choose (3, 3) (30).

We used the term “division” for the VOI condition but the term “allocation” for the Social condition to differentiate. For example, the unequal division (3, 1) behind the VOI would be spun; hence, the DM did not know how much it would receive. On the other hand, the advantageous allocation (3, 1) in the Ad subcondition would not be spun; hence, the DM received 3.

The two allocations, (3, 1) and (3, 3), were chosen for the following reason. We wanted to understand why the equal division was chosen more often behind the VOI, that was, why the unequal division was chosen less often. When the unequal division was selected and spun so that the DM was advantaged, the outcome was the DM received 3 and the RM received 1. We therefore made the advantageous allocation the same as this outcome. Hence, the advantageous allocation was (3, 1). To ensure that the DM received the same in both allocations such that self-interest had no role, the DM had to receive 3 units in the equal allocation. Finally, equity implied the RM should also receive 3. The equal allocation was therefore set to (3, 3).

#### Disadvantageous inequity

In the Dis subcondition, the DM chose between an equal allocation (1, 1) and a disadvantageous one (1, 3). The DM received 1 unit in both allocations and only decided whether the RM received 1 or 3 units (Fig. 2B). This subcondition measured disadvantageous inequity to indicate how averse the DM was to falling behind the RM (30). The two allocations (1, 1) and (1, 3) were designed with an analogous logic.

We also ran two Control conditions, AdC and DisC. The capital letter *C* stood for Control. AdC was the control experiment for the Ad subcondition and was exactly the same as Ad, but with no RM present. In this case, the DM received 3 units no matter which choice it made (Fig. 2C). Likewise, DisC was exactly the same as Dis, except with no RM present. The DM therefore received 1 unit, regardless of its choice (Fig. 2D).

These controls measured the idiosyncratic choices of each DM when alone and faced with two allocations. Because the DM received the same in both allocations, we predicted that the choice frequency would not differ from 50%. This was verified, as described in the “Supplementary Material” section. Each DM–RM pair in the Social condition or each DM in the Control condition was tested via 16 trials carried out over 2 d.

#### Monkeys were averse to getting ahead

We summarized the two directions of inequity concerns first though they were not our main research interest. For advantageous inequity, on average, DMs chose the equal allocation (3, 3) 55.59% of the time in the Ad condition (SD = 9.29%) and 45.31% of the time in the AdC condition (SD = 3.13%). In other words, DMs chose the equal allocation about 11% more often when an RM was present.

To test whether the difference was significant, we calculated the strength of the advantageous inequity concerns. For each pair, we took the frequency with which the equal allocation was chosen in the Ad condition and subtracted the corresponding frequency by the DM in the AdC condition. This frequency difference marked the strength of advantageous inequity concerns. A positive strength meant that the DM chose the equal allocation more often when the RM was present. This showed an aversion to getting ahead. A negative strength meant that the DM liked to get ahead.

**Fig. 3.**
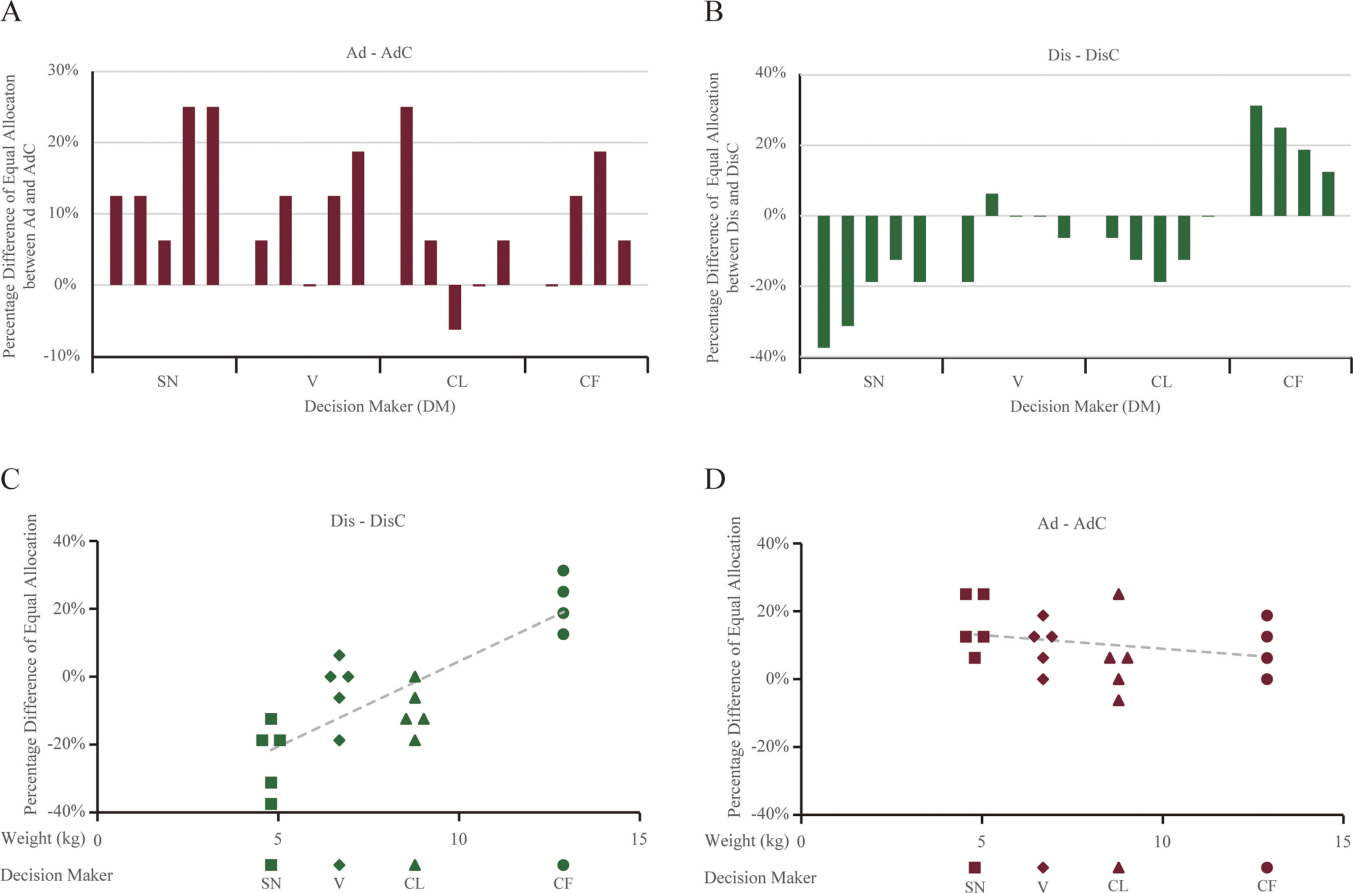
Advantageous and disadvantageous other-regarding concerns. (A) Pair-by-pair breakdown of the Ad versus AdC choices. The figure showed the choice percentage difference of the equal allocation between Ad and AdC. Each bar reflected the strength of advantageous other-regarding concerns. In most pairs, we found a positive strength, consistent with the aversion to getting ahead. To facilitate the comparison, results for advantageous other-regarding concerns were all shown in dark red. (B) Pair-by-pair breakdown of the Dis versus DisC choices. This figure showed that both positive strengths (consistent with the aversion to falling behind) and negative strengths (consistent with loving to fall behind) were found. The DMs were ordered so that the body weight increased from left to right. The tendency for bars to become more positive from left (the lightest DM) to right (the heaviest DM) suggested that body weight might explain individual differences. Results on disadvantageous other-regarding concerns were all shown in dark green. (C) Body weight versus disadvantageous other-regarding concerns. There was a positive correlation between the body weight of the DM and the strength of disadvantageous other-regarding concerns. Heavier monkeys showed greater strength, consistent with a stronger aversion to falling behind. Distinct symbols were used for each DM to help visualize the choices of each DM. Some data points were jittered horizontally to make them all visible. (D) Body weight versus advantageous other-regarding concerns. There was no correlation between the body weight of the DM and the strength of advantageous other-regarding concerns. Some data points were jittered horizontally to make them all visible.

We found that DMs in most pairs (15 out of 19) exhibited a positive strength (Fig. 3A). In a two-level random intercept model, the intercept, estimating the average strength, was positive and significantly different from zero (intercept = 0.11, *z* score = 5.18, *P* < 0.001). Hence, monkeys chose the equal allocation more often when an RM was present. This was consistent with an aversion to getting ahead. We noted that strictly speaking, this was also consistent with prosociality, i.e. the DM would like both the RM and itself to receive more jointly, or an altruistic orientation, i.e. the DM cared about the RM’s payoff. However, even though prosociality or an altruistic orientation could not be ruled out here when we focused on the Ad subcondition, we would argue that it was unlikely when we linked the strength to VOI choices. We would come back to this point when we discussed our results.

#### Aversion to falling behind varied by individual

For disadvantageous inequity, on average, DMs chose the equal allocation (1, 1) 48.68% of the time in the Dis condition (SD = 10.74%) and 53.13% of the time in the DisC condition (SD = 16.54%).

We similarly calculated the strength of disadvantageous inequity concerns. Positive strength meant that the DM chose the equal allocation more often when an RM was present, indicating an aversion to falling behind. Negative strength indicated a preference for falling behind. We found mixed evidence. On the one hand, the DMs in 5 pairs exhibited positive strength, but on the other hand, DMs in 11 pairs exhibited negative strength (Fig. 3B).

At face value, no consistent tendency was evident when an RM was present. This was also reflected by the intercept in a two-level random intercept model. The strength was not significantly different from zero (intercept = −0.04, *z* score = −0.49, *P* = 0.627). However, an interesting pattern was seen in Fig. 3B, namely that choices within each DM seemed to be similar, whereas choices across DMs were different (see bars within each DM and bars across DMs). This suggested that the inconsistency was due to individual differences. Moreover, the strength seemed to increase from left (the lightest DM) to right (the heaviest DM), so we therefore looked into individual differences.

#### Heavier monkeys were more averse to falling behind

If individuals differed in their responses when being disadvantaged, this could explain why we observed inconsistent disadvantageous inequity concerns. Because heavier animals might seldom get less food than others, we tested whether body weight explained disadvantageous inequity concerns. Indeed, we found that heavier monkeys showed a stronger aversion to falling behind (Fig. 3C).

We regressed the strength of disadvantageous inequity on the body weight of the DM in a two-level linear mixed model. The mixed model was similar to the random intercept model, except that body weight was added as a regressor. The regression coefficient associated with the body weight was 0.05 (*z* score = 4.76, *P* < 0.001), significantly different from zero. This meant that a DM that was 1 kg heavier tended to choose the equal allocation (1, 1) 5% more often. The weight difference between the heaviest and the lightest DMs in our experiment was about 8 kg. This would translate to an increased 40% rate of choices of the equal allocation for the heaviest DM than the lightest one. Hence, individually varying disadvantageous inequity aversion might have been due to differences in the DM’s body weight.

This finding that body weight could account for disadvantageous inequity concerns among DMs did not apply to advantageous inequity. A similar regression for advantageous inequity had a slope of −0.01, which was not significantly different from zero (*z* score = −1.19, *P* = 0.235). Thus, lighter monkeys did not appear particularly averse to getting ahead (Fig. 3D). The aversion was consistent and did not vary among animals.

### Step 3: linking choices in front of the VOI with those behind the VOI

After summarizing the two directions of inequity concerns, we returned to our main research interest. We found strong evidence in favor of impartiality, as the monkeys chose the equal division more often behind the VOI. We also found a consistent advantageous inequity aversion and individually varying disadvantageous inequity aversion. We now turned to the empirical question whether inequity aversion was the reason that we observed impartiality behind the VOI.

#### Disadvantageous inequity aversion correlated with impartiality

**Fig. 4.**
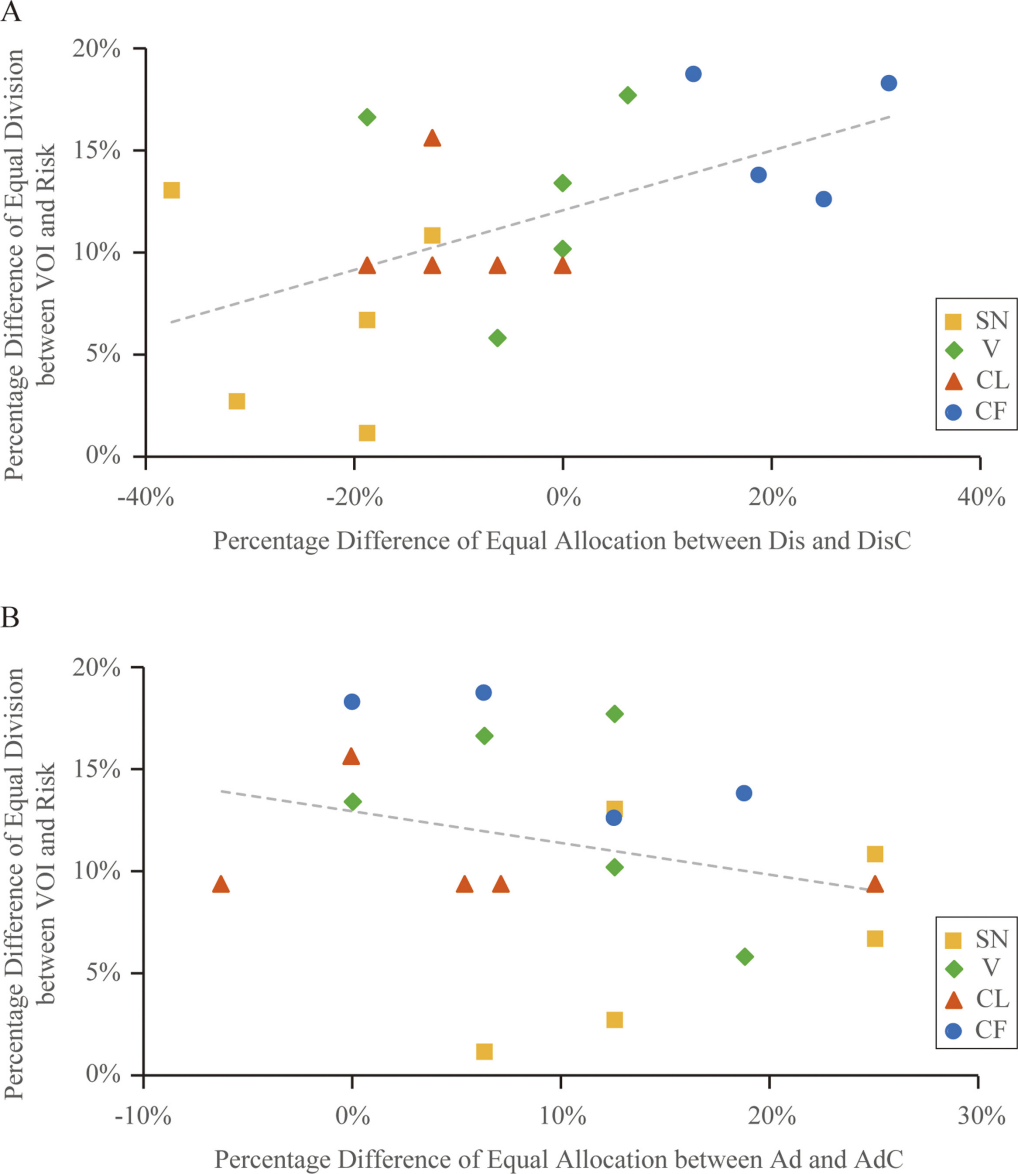
Inequity and impartiality. (A) Disadvantageous other-regarding concerns versus impartiality. There was a positive correlation between the strength of disadvantageous other-regarding concerns and the degree of impartiality. Distinct colored symbols were used for each DM to help visualize the choices of each DM. A DM showing a stronger aversion to being disadvantaged was more impartial. The choices of each DM were close together, suggesting that the correlation might be driven by individual differences. The inset showed the colored symbols for each DM monkey. (B) Advantageous other-regarding concerns versus impartiality. There was no correlation between the strength of advantageous other-regarding concerns and the degree of impartiality. Some data points were jittered horizontally to allow each point to be visible.

We addressed this empirical question by testing whether there was a correlation between inequity and VOI choices. We found a positive correlation between the strength of disadvantageous inequity concerns and the degree of impartiality behind the VOI. In other words, if a DM in a pair was more averse to falling behind, it also chose the equal division more often behind the VOI (Fig. 4A). In contrast, there was no correlation between the strength of advantageous inequity concerns and VOI choices (Fig. 4B).

Formally, we regressed the degree of impartiality on the strength of disadvantageous inequity concerns. In a two-level linear mixed model, the regression coefficient was 0.15, significantly different from zero (*z* score = 2.72, *P* = 0.006). This indicated that if a DM in a pair exhibited a 1% greater strength of disadvantageous inequity concerns, it chose the equal division 0.15% more often behind the VOI. Continuing our numerical comparison using the heaviest and lightest DMs, a 40% greater strength of disadvantageous inequity concerns predicted a 6% greater chance of equal division behind the VOI. A similar regression on advantageous inequity was not significant. The coefficient was −0.11 (*z* score = −0.99, *P* = 0.324). We verified that these two correlations were indeed different statistically. To do that, we regressed the degree of impartiality on the strengths of disadvantageous inequity concerns and advantageous inequity concerns in one two-level linear mixed model. The regression coefficient of disadvantageous inequity concerns was 0.14 (*z* score = 2.73, *P* = 0.006) and that of advantageous inequity concerns was −0.13 (*z* score = −1.28, *P* = 0.200). The chi-squared statistics on whether the two coefficients were the same was significantly different from zero [chi^2^ (*df* = 1) = 5.97, *P* = 0.0145]. This showed the dissociation between these two directions of inequity concerns.

Together, these pointed to the importance of disadvantageous inequity, a particular direction of inequity in front of the VOI, in explaining why monkeys appeared impartial behind the VOI. When the aversion to falling behind was stronger, we observed an increased tendency to be impartial behind the VOI. This could arise because when the DM was behind the VOI, it could not guarantee its own advantage. If the aversion to being disadvantaged was stronger, the DM might divide the resources more equally ex ante to prevent itself from being disadvantaged ex post. Hence, it appeared impartial.

Furthermore, we could see that in Fig. 4A, the choices of each DM (as represented by DM-specific symbols) were close together. This suggested that the correlation between disadvantageous inequity and impartiality could be driven by individual differences. We examined this possibility in the next step.

### Step 4: a possible underlying factor for the linkage

We observed a link between disadvantageous inequity concerns and impartiality in step 3. Because there was a correlation between body weight and disadvantageous inequity concerns as shown in step 2, one might wonder whether body weight was the reason for the link in step 3. To address this, we ran two exploratory analyses in the “Supplementary Material” section. Results were consistent with this post-hoc hypothesis. We showed that the body-weight predicted part of disadvantageous inequity concerns correlated with the degree of impartiality behind the VOI. However, after removing this body-weight predicted part, the residual disadvantageous inequity concerns no longer correlated with the degree of impartiality. These results suggested that it was probably not disadvantageous inequity concerns per se that mattered. Instead, the key was the disadvantageous inequity concerns predicted by body weight. Hence, we conjectured that body weight could be an important factor for the link between disadvantageous inequity concerns and impartiality.

## Discussion

We study Formosan macaques’ choices behind the VOI. We find that the monkeys choose the equal division of resources more often behind the VOI than in the Risk condition. This suggests impartiality. To understand impartiality better, we also study choices in front of the VOI, where we observe consistent advantageous inequity aversion and individually varying disadvantageous inequity aversion. The latter has an important role because the degree of impartiality is linked to this individually varying inequity aversion. Moreover, the body weight of each individual correlates with disadvantageous inequity and is likely the reason that we see a link between impartiality and inequity. The most parsimonious explanation of the linkage is as follows. Heavier monkeys are more averse to disadvantageous inequity. Because there is no way to guarantee how much a DM will receive behind the VOI, a stronger aversion to being disadvantaged makes it choose the equal division more often. This suggests that impartiality may be based on a primitive negative reaction to being disadvantaged. Thus, metaphysical reasoning may not be a necessary condition for precursory morality, including the impartial behavior studied here, in other nonhuman species.

Because we compare the presence of an RM with its absence, an alternative explanation of our results is that the DMs are motivated by the presence of an RM, consistent with the social facilitation effect. However, this alternative explanation does not predict a specific directional effect. That is, it is not clear whether a more motivated DM would choose the equal division more often or less often behind the VOI. Hence, this is unlikely to explain our results. To further address this alternative explanation directly, we test whether the DMs are indeed differentially motivated, depending on whether an RM is present in the “Supplementary Material” section. We examine two behaviors, namely bar pull failures and food refusals, which are often used to measure motivation. Monkeys rarely fail to pull a bar or reject food. The frequencies in the presence or the absence of an RM are not different. Hence, the direct measurement of motivation does not support the social facilitation explanation. Perhaps this is because grapes, the rewards provided in this experiment, are highly valuable. In the literature, a “distraction control” is sometimes used, in which an RM is present but denied access to rewards. The purpose is to control for the social facilitation effect. Because we do not find evidence for the social facilitation effect, our use of the absence of an RM as the control is justified.

Chimpanzees exhibit a prepotent bias, according to which they tend to choose the option that has a greater amount of rewards (15). This bias may be thought to have influenced our results. Because both the equal division (2, 2) and the unequal division (3, 1) total 4, this bias has no role in the Risk or VOI conditions. In front of the VOI, we have clear evidence against such a bias. In the AdC condition, the option with a greater reward (3, 3) is chosen only 45.31% of the time. In the DisC condition, the option with a greater reward (1, 3) is chosen 46.87% of the time. Therefore, this bias cannot explain our results.

Formosan macaques are genetically and ecologically most similar to rhesus macaques and Japanese macaques (31, 32). They eat fruits and leaves mostly (33). Because females stay with the social group, they have stable matrilineal hierarchies (34, 35). The fusion–fission rate is not high in their despotic society (36, 37). Previous studies have found evidence of inequity aversion in rhesus macaques (24) and Japanese macaques (20). These are largely consistent with our findings. Moreover, similar to a report in capuchin monkeys (14), our monkeys are not that averse to falling behind, but some are averse to getting ahead. The range of cooperation behavior observed in Formosan macaques may provide a hint of this. Formosan macaques emit food calls to share information on the presence of food (38). They are also observed to co-feed as a group on a food patch and agonistic events do not increase with the number of individuals feeding on that patch (39). They allomother unrelated infants as frequently as kin-related infants (40). These cooperative behaviors, together with the evidence that larger groups have an advantage in inter-group competition (35), suggest that their capacity to cooperate may have been selected for due to the need to communally defend against other groups. Within a cohesive social structure (41), individuals are likely to be less competitive. This may make them react less strongly to disadvantageous inequity, and in a similar vein, avoid seeking to get ahead.

We show that body weight correlates with disadvantageous inequity aversion and speculate that this is the reason that we see a link between impartiality and inequity. Because body weight is the best predictor of social rank (42–44), and rank often correlates with behaviors (22, 23), one may wonder whether results would be similar if we replace weight with rank. In the “Supplementary Material” section, we assign an ordinal rank to our monkeys and analyze its effect. Step 2 holds up if we replace weight with rank: The assigned ordinal rank still correlates with the disadvantageous inequity aversion. That is, higher-ranked monkeys are more averse to being disadvantaged. However, we do not find full support for the conclusion that rank is the reason for the link between impartiality and inequity. The difference in the results for weight and rank could be due to either or both of two reasons. First, weight is a trait marker, but rank is a state marker. A trait marker captures physiological differences, such as aggressive responses, more directly, whereas a state marker better reflects the influence of social structure. If the underlying mechanism is physiological, the trait maker may have better explanatory power. For example, weight but not rank correlates with the level of testosterone in cynomolgus monkeys (45). Second, the assigned ordinal rank may be an imprecise measure of rank. Our data cannot provide further evidence. For completeness, we further look into other factors, including weight difference, assigned ordinal rank difference, gender, gender difference, or whether two monkeys were housed in the same cage. None of them can explain disadvantageous or advantageous inequity aversion. Our conclusion stays the same. Therefore, body weight plays an important role in our data. However, the speculation that body weight could be the reason for the link between impartiality and inequity should be treated with caution. Clarification of the role of body weight would still require future research.

An alternative interpretation for the fact that some monkeys choose the equal allocation (3, 3) in the Ad condition and the disadvantageous allocation (1, 3) in the Dis condition is prosociality or an altruistic orientation. If prosociality drives our result for conditions in front of the VOI, when the DM is present, the DM prefers the RM and itself to receive a larger total. This interpretation seems as adequate as the inequity interpretation when we consider the Ad or Dis condition alone. However, when we link these choices in front of the VOI conditions to the behind the VOI choices, the interpretation based on prosocial motives seems unlikely. Both the equal division (2, 2) and the unequal division (3,1) give the DM and RM 4 in total, so a prosocial DM would not find the equal division more prosocial than the unequal division. Therefore, it cannot explain why the equal division is chosen more often. Similarly, an altruistic-oriented DM would find the equal division (2, 2) gives 2 units to the RM and the unequal division (3, 1) gives the RM 2 units on average. It is not obvious whether an altruistic-oriented DM will favor the equal division or the unequal division. We thus interpret the link as a more inequity-averse DM showing a higher degree of impartiality behind the VOI. This is a benefit of studying a suite of choices. Doing so allows access to a range of behaviors that are more informative than a single choice behavior. Thus, certain interpretations become unlikely when we study multiple choice behaviors and seek to explain them altogether parsimoniously.

Studying a suite of choice behaviors may also shed new light on the debate whether inequity aversion exists in nonhuman primates. The existence of a behavior means animals show this behavior consistently. In our case, disadvantageous inequity aversion varies across individuals, so we do not find evidence for this direction of inequity aversion. However, by studying the VOI choices at the same time, we find a correlation between the VOI choices and disadvantageous inequity aversion. This suggests a role for disadvantageous inequity aversion that can only be appreciated when the VOI choices are also examined. In other words, an inconsistent behavior may still be important. Individual differences may average out so that the mean across individuals is close to zero and is therefore concluded as inconsistent. However, using a new behavioral assay, if a correlation is found between these individual differences (in our case, the strength of disadvantageous inequity aversion) and the behaviors in the new assay (the degree of impartiality), the early inconsistency in the results may be important. By focusing only on consistency, we may miss the importance of meaningful inconsistent behaviors.

In the “Supplementary Material” section, we rule out the possibility that the DM and RM reciprocate each other, on top of the extra care we take to avoid this possibility when we run the experiment. Because we run conditions with no RM before their corresponding conditions with an RM, the order effect could be a potential confound. We describe the details in the “Supplementary Material” section and explain why it may not be serious. We further address the essence of the order effect, the time effect, in the “Supplementary Material” section. We should note, however, that the order effect cannot be ruled out, despite that in a human VOI experiment (46), by carrying out the experiment in different orders, no order effect was found. Another limitation of our study is the sample size. Though we have exhausted all the possible pairs in the lab, the limited sample size may make it difficult to generalize our results to the entire species. In that case, our results can be read as what Formosan macaques are capable of. We further supplement our results with nonparametric analyses in the “Supplementary Material” section. All the results are robust and our conclusion stays the same.

Our results have implications for cooperation (7). Because there is always a chance of being disadvantaged behind the VOI, if the aversion to falling behind is strong, a DM may divide the resources more equally to prevent that from happening. Seemingly impartial divisions are made when a DM is behind the VOI and therefore unable to guarantee its own advantage. This, in turn, may support cooperation in the case of a risk. In a natural setting, such as in collaborative foraging, no one can know beforehand who will eventually be advantaged. However, animals may learn to divide opportunity equally in advance. In turn, this makes continuing cooperation likely. This resembles what is captured by the concept of the VOI. Moral behavior in humans is certainly much more elaborate and complex (47–49). In other species, pervasive aversion to falling behind may first evolve due to pressure to survive. However, this could also promote an equal division, making risky cooperation possible (7). We speculate that this channel helps develop precursory forms of morality, echoing the Darwinian notion that morality is based on cooperation (1).

## Funding

This work was supported by the Ministry of Science and Technology of Taiwan (grant numbers 103-2410-H-002-008-MY3, 104-2320-B-002-065-MY3, and 109-2320-B-002-014-MY3).

## Authors' Contributions

Y.T.L.: designed and performed research. W.-H.H.: performed research and analyzed data. Y.-T.H.: performed research and analyzed data. T.-Y.H.: performed research and analyzed data. J.-D.Z.: analyzed data. C.-I.Y.: designed research, contributed new reagents/analytic tools, and analyzed data. C.-Y.H.: designed research, analyzed data, and wrote the paper.

## Supplementary Material

pgac188_Supplemental_Files

## Data Availability

All data can be found at https://www.space.ntu.edu.tw/navigate/s/66ACE28E2C794C2B84E6CE2B52724632QQY.
