# Supplementary material for: Choices behind the veil of ignorance in Formosan macaques
Source: PNAS Nexus. 2022 Sep 15;1(4):pgac188. doi: 10.1093/pnasnexus/pgac188 (PMC9802069; doi:10.1093/pnasnexus/pgac188)
Supplement: pgac188_Supplemental_Files [file pgac188_supplemental_files.zip › PNASNEXUS-PNASNEXUS-2021-00249-s01.docx]

Supplementary Materials

**This PDF file includes:**

Materials and Methods

Table S1

Supplementary Text

Captions for Movies S1 and S2

**Other Supplementary Materials for this manuscript include the following:**

Movies S1 and S2

**Materials and Methods**

**Apparatus**

Six monkeys were each familiarized with the experimental apparatus. The apparatus had two sliding trays, upper and lower. Each tray was connected to a pull bar. The upper bar was connected to the upper tray, and the lower bar was connected to the lower tray. The monkeys made choices by pulling one of the two bars. Once a bar was pulled, the other bar was locked and could no longer be pulled. Each tray had two food dishes, one to the left and one to the right.

The monkeys were in a quadrant cage during the experiment. We used two compartments, one to the left and one to the right. The DM was always in the left compartment for each decision. Before making the choice, the two dishes on each tray faced the DM. Once a choice was made, the selected tray was slid forward at an angle so that the left dish was only accessible from the left compartment, and the right dish was only accessible from the right compartment.

In different experimental conditions, a passive RM may or may not be present in the right compartment, and the chosen tray may or may not be spun. The door between the left and right compartments was always closed unless otherwise noted.

**Pretests**

Four pretests were run with each DM alone before the main experiment. Because our major interest was in the distribution of rewards between the DM and RM, we made sure that the DM was able to attend to the dishes on its side and to the side of the RM. We designed pretests 1-3 for this purpose. Step by step, pretest 1 was performed to ensure that the DM was attending to the dish on its own side. Pretest 2 was done to make sure that it attended to the dishes on RM’s side. Pretest 3 was conducted to ensure that the monkey attended to both sides. To implement the VOI condition, we introduced spinning after pretest 3. Pretest 4 was intended to make sure that the monkey understood that the selected choice would be spun.

**Pretest passing standard**

Each pretest had 5 blocks. There were 12 trials in each block. A DM had to select the choice with the higher (expected) amount of rewards in at least 8 trials to pass a block. It had to pass at least 3 blocks to pass a pretest. The probability of a randomly behaving DM passing a pretest was 5%.

Our passing standard was different from the usual standard in the literature, in which a stricter standard was imposed to pass each block (such as 5 passes out of 6 trials or 10 passes out of 12 trials in a block). However, in the usual standard, the number of blocks allowed was not capped in advance. Because the number of blocks was not capped, the probability of passing a pretest using the usual standard was not known in advance. We chose our standard because we hoped to control the probability of passing a pretest in advance. In fact, when passing a block, 73% of the time monkeys chose the higher reward option more than 8 out of 12 times, the cutoff criterion that we used. When passing a pretest, 75% of the time, monkeys took less than 5 blocks, the maximum number of blocks that we allowed. These were taken as indications that the monkeys understood the experimental procedure.

**Notation for the baited content**

In the pretests and the main experiment, the reward unit was one-eighth of a grape. This was done to prevent the monkeys from losing motivation due to consuming too many grapes in a given experimental day. Moreover, they were hungry during the experiment, as a half-portion of the meal was given only after the experiment was complete. For ease of exposition, if a tray was baited with x units in one dish and y units in the other, this was notated by (x, y). In other words, we only referred to the baited content of a choice. When the position also mattered, i.e., whether x was in the left or the right dishes, we stated that in words.

**Pretest 1: (3, 0) vs. (2, 0)**

In this pretest, one of the two left dishes was baited with 3 units and the other with 2. We counterbalanced whether the upper or the lower tray was baited with (3, 0). Choice displays were always counterbalanced whenever possible. Hence, this was not explicitly referred to again later on. A monkey was tested whether it would choose (3, 0). This was to show that the DM could reliably pay attention to the dishes on its side.

**Pretest 2: (0, 3) vs. (0, 2)**

In this pretest, one of the two right dishes was baited with 3 units and the other with 2. The door between the two compartments was open so that, after making a choice in the left compartment, a monkey could go to the right compartment to obtain the rewards. A monkey was tested whether it would choose (0, 3). This aimed to show that the DM could reliably pay attention to the dishes on the side of the RM.

**Pretest 3: (1, 2) vs. (1, 1)**

In this pretest, the two left dishes were both baited with 1 unit. One of the two right dishes was baited with 2 units and the other with 1. The door between the two compartments was open so that, after making a choice in the left compartment, a monkey could also go to the right compartment to obtain the rewards. A monkey was tested whether it would choose (1, 2). This was to ensure that the monkeys did not pass pretest 2 because of the omission of food on its side and that the DM was able to pay attention to the dishes on both sides.

**Spinning**

We spun the selected tray after pretest 3 to prepare the monkeys for the VOI/Risk condition. The spinning was carried out as follows. Half of the time, the selected tray was spun one loop so that the left dish remained on the left. The other half of the time, it was spun one and a half loops so the right dish came to rest on the left. If an (x, y) tray was selected, because the DM was in the left compartment and could only access what was spun to the left, it amounted to receiving x half of the time and receiving y half of the time.

The monkeys went through many trials after pretest 3 to familiarize them with the spinning. After the familiarization trials, we proceeded to pretest 4. Because the familiarization trials were crucial in ensuring the monkeys understood the spinning, we described them in detail. The content of these familiarization trials varied. One key principle was that the monkeys had never pre-experienced pretest 4 or any VOI/Risk situation in which the spinning was carried out. The other principle was the familiarization trials went from easy to difficult to help monkeys learn gradually.

Because the DM could only access what was on the left, when the selected tray was not spun, only the left dish mattered. But when it was spun, the initial baiting position became irrelevant, as the right dish could also come to rest on the left. Hence, the familiarization trials were designed to ensure that the monkeys could understand that the initial baiting position was irrelevant due to spinning. We designed three types of trials to familiarize monkeys with this.

With spinning, because both the left and right dishes could come to rest on the left, a DM should take both into account. Suppose the optimal choice in a spinning case was called tray A. In the first type of trials, only one tray was baited with rewards. Hence even if a monkey focused on the left, by coincidence it chose tray A because only A had rewards. In the second type, we baited the two left dishes with the same amount of reward. Hence, if a monkey focused on the left, it would be indifferent and chose A half of the time. In the third type, we baited tray A with a smaller reward on the left. Hence, a monkey focusing on the left would not choose A. Because a monkey focusing on the left would choose A less and less likely from the first type of trials to the third type of trials, the first type was easier than the second, which was in turn easier than the third. Therefore, generally, the first type proceeded before the second, which proceeded before the third. Depending on how well the monkey learned, within a given type, we progressed to make the trials gradually more difficult.

The first type was denoted by (L, R), (0, 0). Tray A was baited with L on the left and R on the right, but the other tray was baited with nothing. This type was easy, as tray A was the obvious choice. Indeed, the monkeys chose tray A 90.18% of the time. The purpose of this type was to allow the monkeys to experience the consequence of spinning when they were not yet familiar with it.

The second type was denoted by (L, R*), (L, R). Both trays were baited with rewards L on the left. On the right, one tray was baited with R* and the other with R where R*>R. Hence tray A was (L, R*). This type was less easy because taking only what was on the left into account, the two trays were equally good. However, if the monkeys understood the selected tray would be spun, they would consider A to be better. During the first 30 trials, the DM’s choice of A ranged from 17 to 19 times. This was not significantly different from 50% for every DM (the smallest p-value among all DMs was 0.2000). This showed that initially the monkeys were unaffected by what was baited on the right despite having experienced spinning from trials of the first type.

In the third type, one tray had a larger reward on the left. However, this tray in fact was dominated by the other tray when one understood that spinning made the initial baiting position irrelevant. This type was denoted by (L*, R), (L, R*), where one tray had L* on the left, R on the right; the other tray had L on the left, R* on the right. We made L*>L. Hence, by focusing on the left, (L*, R) seemed to be the better choice. However, we made R* large and R small, such that R*>L* and L>R. Hence, if one ignored the initial baiting position, the (L, R*) tray actually dominated the (L*, R) tray. This type really required the monkeys to understand that spinning made the initial baiting positions irrelevant. Had they kept focusing on the left, they would miss the possible large reward R* and would likely receive the small reward R. As expected, this type of trial was not easy for the monkeys. However, they did learn over time. Because we made trials progressively more difficult depending on how well the monkeys learned, we compared the first 30 trials to the last 30 trials of the same difficulty. Overall, during the first 30 trials, the monkeys chose the dominating tray only 53.0% of the time. The frequency increased by 20.2% to 73.2% of the time in the last 30 trials. This increment was significant (z score = 4.19, p = 0.000). After these familiarization trials, we proceeded to pretest 4.

**Pretest 4 (2, 4) vs. (3, 1)**

This pretest aimed to show that monkeys understood spinning. One tray was baited with 2 units in the left dish and 4 in the right dish. The other tray was baited with 3 in the left dish and 1 in the right dish. If a monkey did not understand spinning and focused on the two left dishes, then a rational response was to choose (3, 1), because 3 was better than 2. On the other hand, if it understood spinning made the initial baiting position irrelevant and both could be spun to the left, then since the bigger reward of the (2, 4) tray was greater than that of the (3, 1) tray (i.e., 4 > 3) and the smaller reward of the (2, 4) tray was also greater than that of the (3, 1) tray (i.e., 2 > 1), it should choose (2, 4). This pretest, like the third type, tested the essence of spinning. However, the monkeys had never experienced this pretest before. It was new to them.

**Explanations on why monkeys did not pass pretest 4 because they preferred a greater sum of rewards**

Chimpanzees were found to exhibit a prepotent bias that they were biased toward the option with a greater sum of rewards (15). Because the (2, 4) tray had a greater sum than the (3, 1) tray, a DM might have passed pretest 4 due to this bias instead of having understood spinning.

This possibility was unlikely in our data for the following reasons.

First, if a DM preferred the tray with a greater sum, at the early stage of type 2 familiarization trials, no matter whether a DM had understood spinning or not, it should choose the (L, R*) tray more often than the (L, R) tray. This was because if a DM did not understand spinning, this preference for a greater sum should lead to the choice of (L, R*). On the other hand, if a DM understood spinning, it had an additional reason to choose (L, R*), as spinning made R* accessible half of the time. But as stated above, this was not the case. None of the DM’s choice of the (L, R*) tray was significantly different from 50% during the first 30 trials. This showed that monkeys at this stage were not yet affected by the dishes on the right side.

Second, choices in the AdC and DisC conditions did not support that monkeys preferred the tray with a greater sum either. In the AdC condition, a DM chose between (3, 3) and (3, 1). If it preferred the tray with a greater sum, it would choose (3, 3). However, (3, 3) was chosen only 45.31% of the time. In the DisC condition, a DM chose between (1, 1) and (1, 3). This prepotent bias predicted the choice of (1, 3). However, it was chosen 46.87% of the time. None of the DM’s choice was significantly different from 50%, consistent with the conclusion that the monkeys were not affected by the inaccessible right dishes. This was shown in the bottom panel of Table S1.

Type 2 trials were done before pretest 4. The AdC and DisC conditions were part of the main experiment and therefore were run after pretest 4. Hence, we did not find evidence that monkeys preferred the tray with a greater sum either before or after pretest 4. This bias was unlikely a concern in our data.

There were 6 monkeys in the lab. We did not make any selection, so all of them went through the same procedures. Two monkeys did not pass the pretests and could only act as RM. We had at most (6 - 2) * (6 - 1) = 20 DM-RM pairs. However, two monkeys attempted to fight through the dividing mesh when they were paired, so we were unable to pair them in the experiment. One of them failed the pretests, leaving 20 – 1 = 19 DM-RM pairs. We exhausted all 19 possible pairs.

**The main experiment**

The main experiment had four conditions: Risk, VOI, Control and Social. In a human experiment (46), by carrying out the experiment in different orders, no order effect was found: whether the VOI condition was run first or second did not matter. Based on this, we ran the conditions with no RM before the conditions with an RM. The Risk condition was run first, then the VOI condition, followed by the Control condition and finally the Social condition. The order of the AdC and DisC conditions was counterbalanced across subjects. Similarly, the order of the Ad and Dis conditions was counterbalanced across subjects.

**Order effect**

Because we did not counterbalance the order of conditions, in principle, the order effect could be a confounding factor. However, our data provided some evidence that it was likely not a serious concern.

1. **Choices were consistent with a clear preference or self-interest**

First, one possible reason that order might have had an effect was due to learning. If monkeys learned over time, there might be the concern that choices in the conditions with no RM were random because they were run earlier, and learning might not have been adequately completed. However, in the Risk condition, DMs clearly showed a preference for risk instead of choosing randomly. Each individual’s choice frequency ranged from 15.63% to 28.13%, all consistent with risk-loving. We used the binomial test to examine whether the choice frequency of each monkey equaled 50%. All DMs’ choice frequency was significantly different from 50%. In the AdC and DisC conditions, because the DM received the same amount in both allocations, the choice frequency should not have differed from 50%. This was indeed the case. None of the DM’s choice was significantly different from 50%. These tests were in the bottom panel in Table S1. In all three conditions with no RM, every DM’s choices were consistent with a clear preference or self-interest. Hence, their choices were not likely to be the result of not having learned well.

1. **The essence of the order effect: Time effect**

Second, we examined the order effect, taking the risk and VOI conditions as an example. If there were an order effect, whether the Risk condition was run before or after the VOI condition would be important. This could happen when a condition run earlier in time differed from the same condition run later in time. Although our data could not directly address the order effect, we could address the essence of it, namely, the time effect, indicating whether we observed choice in the same condition changed as time went. We did not find any such evidence. This was shown in section (6) Time Effect in **Supplementary Text**.

These two pieces of evidence indicated that the order effect was likely not serious.

**Details to avoid reciprocation**

Each condition was divided into blocks of 8 trials. For instance, there were 32 trials for each DM-RM pair in the VOI condition. Because we only ran one block on any given day, this meant that each DM-RM pair met over four days. To minimize the possibility of reciprocation, we ran the experiment with the following three restrictions. First, there were at least three days before any two monkeys met again. Second, a monkey could only act as a DM once a day. In that situation, it could at best act as an RM once later on the same day. Third, if a monkey first acted as an RM on one day, it could not act as a DM on the same day. At most, it could act as an RM twice in one day.

**Ethics statement**

The monkeys resided in quadrant home cages similar to the experimental cage. Two monkeys were housed in each cage but were always separated by a dividing mesh and could not interact with each other directly. The experiment was approved by the Council of Agriculture, Executive Yuan, Taiwan, and the Institutional Animal Care and Use Committee of National Yang-Ming University under permit numbers 1010601 and 1050303. All experimental procedures were performed in accordance with the guidelines of the U.S. Department of Agriculture.

**Videotaping**

The experiment was videotaped from two angles: one in the direction of the food trays, recording the available choices, and the other in the direction of the monkeys, recording the DM’s choices. One experimenter coded the DM’s choices. A blind observer coded a random selection of 20.11% of trials by watching the videotapes. Cohen’s kappa was 1, implying complete agreement.

**Table S1** Summary for the Test Results

| **Test conditions** | | **Results** |
| --- | --- | --- |
| **Impartiality** | | |
| Null hypothesis:  VOI = Risk | Options:  (2, 2) vs. (3, 1) | Percentage of choosing the equal division:  VOI: 33.56%, Risk: 22.54%, **p < 0.001**  Monkeys are impartial (Fig. 1B). |
| **Other-regarding concerns** | | |
| Null hypothesis:  Ad = AdC  Dis = DisC | Options:  (3, 3) vs. (3, 1)  (1, 1) vs. (1, 3) | Percentage of choosing the equal allocation:  Ad: 55.59%, AdC: 45.31%, **p < 0.001**  Dis: 48.68%, DisC: 53.13%, p = 0.627  Monkeys show advantageous other-regarding concerns, consistent with the aversion to getting ahead (Fig. 3A, B). |
| **Other-regarding concerns and body weight** | | |
| Correlation between weight and  Ad-AdC  Dis-DisC | | Regression coefficient of weight for  Ad-AdC: -0.01, p = 0.235  Dis-DisC: 0.05, **p < 0.001**  Heavier monkeys show stronger disadvantageous other-regarding concerns, consistent with the stronger aversion to falling behind (Fig. 3C, D). |
| **Impartiality and inequity** | | |
| Correlation between the degree of impartiality (VOI-Risk) and  Ad-AdC  Dis-DisC | | Regressing the degree of impartiality on  Ad-AdC: -0.11, p = 0.324  Dis-DisC: 0.15, **p = 0.006**  A stronger aversion to falling behind correlates with a higher degree of impartiality (Fig. 4A, B). |
| **Examining conditions with no RM using the binomial test** | | |
| Null hypothesis:  Risk = 50%  AdC = 50%  DisC = 50% | Options:  (2, 2) vs. (3, 1)  (3, 3) vs. (3, 1)  (1, 1) vs. (1, 3) | Risk-loving for all DMs (**p < 0.02** for all DMs)  Close to 50% for all DMs (p > 0.8 for all DMs)  Close to 50% for all DMs (p > 0.08 for all DMs) |

The main results are highlighted in red.

**Supplementary Text**

We explained the concept of the two-level regressions and reported several supporting results.

1. Two-level regressions

We used the VOI vs. Risk result to illustrate the two-level regressions that we ran. For each pair in the VOI condition, we took the frequency the equal division was chosen and subtracted the corresponding frequency when the DM was alone in the Risk condition. This difference reflected the degree of impartiality: a higher difference meant that the DM chose the equal division more often in the VOI condition. The dependent variable was this degree of impartiality. Because we had 19 pairs, there were 19 data points. The two-level random intercept model had the first level being the pair and the second level being the DM. If we denoted the dependent variable by VOI-Risk because we calculated the difference, “two-level” implied a need for two subscripts, one for the pair (the first level) and the other for the DM (the second level). Each data point was presented as VOI-Risk_pair i, DM j_, indicating the degree exhibited in pair i by DM j.

The two levels could be spelled out as follows:

(E1) VOI-Risk_pair i, DM j_ = β_DM j_ + ε_pair i, DM j_

(E2) β_DM j_ = γ+μ_DM j_

(E1) meant the degree was determined by who the DM was (the β_DM j_ term) and an error term (the ε_pair i, DM j_ term). (E2) further specified that the β_DM j_ term had a mean across all DMs (the γ term) and a DM-specific random error (the μ_DM j_ term).

If we substituted (E2) into (E1), we got

(E3) VOI-Risk_pair i, DM j_ =γ+μ_DM j_ +ε_pair i, DM j_

From (E3), it could be seen that we used 19 data points to estimate the mean across 4 DMs. Even where 19 data points (one data point for one pair) were present, γ was the average of 4 DMs. In the error structure, each DM had an individual specific random effect μ_DM j_, and there was also a randomness of each data point ε_pair i, DM j_. This meant any two pairs with the same DM j had a shared random term μ_DM j_, so they could be correlated. In other words, the DM was modeled as a random effect to account for the correlation within each DM. Hence, we did not treat the 19 points as independent. When we reported that we found a significant difference between VOI and Risk, we were testing whether the overall intercept γ was significant.

If we instead averaged data points of each DM first and ran regressions using the 4 averaged data points (corresponding to 4 DMs), our results did not change qualitatively. We preferred the two-level regressions because we used as much information as possible.

1. Motivation or arousal

One potential explanation of our findings was that DMs were more motivated/aroused with the presence of an RM than without it. We looked into two behaviors, namely, bar pulls and food refusals, which were often used to measure motivation/arousal. If monkeys were more motivated, they would pull bars more often and refuse food less often. In our experiment, probably because grapes were highly valuable rewards, the frequency of failing to pull a bar or food refusal was very low. We did not find that DMs were more motivated/aroused when an RM was present.

In the Risk condition, the DMs failed to pull a bar only 3.1% of the time. In the VOI condition, the percentage was only 4.4%. The difference was not significant (p = 0.6263) in a two-level random intercept regression. As for food refusals, the DMs never refused foods in the Risk condition and only did so 0.33% of the time in the VOI condition. These two frequencies were not statistically significantly different either (p = 0.3042).

In other conditions (Ad, AdC, Dis, and DisC), DMs never failed to pull a bar or refuse foods. There was thus no need to perform continuing statistical tests. Overall, there was no evidence that DMs were more motivated/aroused when an RM was present.

1. Modulation by body weight

We observed a link between disadvantageous inequity concerns and impartiality in step 3. Because there was a correlation between body weight and disadvantageous inequity concerns as shown in step 2, we wondered whether body weight was the reason that we saw the link in step 3. To show the importance of the body weight for modulating the correlation between disadvantageous inequity concerns and choices behind the VOI, we proceeded with two sets of regressions. In the first set, we showed that the body weight predicted part of the disadvantageous inequity concerns accounted for the impartiality behind the VOI. In the second set, we showed that after removing this body weight predicted part, the residual disadvantageous inequity concerns no longer accounted for the impartiality behind the VOI.

With the first set, we proceeded in two steps. We used a two-level linear mixed model in each step. In the first step, we regressed the strength of disadvantageous inequity concerns (the proportional difference that the equal allocation was chosen between Dis and DisC, denoted as Dis-DisC) on the body weight of the DM to obtain the fitted strength of the disadvantageous inequity concerns (denoted as $\hat{Dis-DisC}$). This was the predicted strength, as predicted by the body weight. Then, in the second step, we regressed the proportional difference that the equal division was chosen between VOI and Risk (denoted as VOI-Risk) on this predicted strength. The coefficient of the predicted strength was 0.18 (z score = 2.89, p = 0.004) and was statistically significant.

Illustratively, we ran

Dis-DisC on body weight to get $\hat{Dis-DisC}$.

Then, we ran

VOI-Risk on $\hat{Dis-DisC}$.

(p = 0.004)

This result indicated that the choice difference between VOI and Risk could be explained by the body weight predicted strength of disadvantageous inequity concerns.

In the second set, we also proceeded in two steps. In the first step, we regressed the strength of disadvantageous inequity concerns on the body weight to obtain the residual (denoted as (Dis-DisC)-$\hat{(Dis-DisC)}$). Then, in the second step, we regressed the proportional difference that the equal division was chosen between VOI and Risk on the body weight and the residual. The coefficient of the body weight was 0.01 (z score = 2.89, p = 0.004), and it was statistically significant. The coefficient of the residual was 0.01 (z score = 0.10, p = 0.924), but it was not significant.

Illustratively, we ran

Dis-DisC on the body weight to get the residual (Dis-DisC)-$\hat{(Dis-DisC}$).

Then, we ran

VOI-Risk on the body weight and the residual (Dis-DisC)-$(\hat{Dis-DisC})$.

(p = 0.004) (p = 0.924)

This result indicated that the choice difference between VOI and risk could not be explained by the residual disadvantageous inequity concerns if we removed what was predicted by the body weight.

These two results together suggested the importance of the body weight. The body-weight predicted strength of disadvantageous inequity concerns accounted for the difference between VOI and Risk. However, the body-weight **un**predicted disadvantageous inequity concerns did not account for the choice difference between VOI and Risk. Hence, the body weight was a key factor in modulating the correlation between the disadvantageous inequity concerns and the impartiality behind the VOI.

1. The body weight, the ordinal rank and other factors

Our monkeys were never in a shared space. Hence, we were unable to observe who took food first and who escaped from whom in the shared space to determine their ranks. However, we did observe some behavior, such as the following: when we fed them, whether feeding a particular animal before another would arouse the latter and whether moving the cage an animal was in during housekeeping would elicit some display of the other. There was a consensus among the animal keepers and experimenters on the ranks. If we assigned ordinal ranks to our six monkeys arguably arbitrarily according to this consensus, such that the highest-ranked animal got 6 and the lowest-ranked animal got 1, the Pearson correlation coefficient between the body weight and the assigned ordinal rank among the DMs was 0.915, which was high.

The assigned ordinal rank was subjective, and the body weight measure was objective. Hence, we reported the body weight result in the main text. We also tested whether the results on the body weight still held if we replaced the body weight with the assigned ordinal rank. Recall that the regression of the strength of disadvantageous inequity concerns on the body weight of the DM had a positive slope of 0.05 (z score = 4.76, p < 0.001). When we replaced the body weight by the ordinal rank, the slope was again positive at 0.07 (z score = 2.25, p = 0.025) and still significant. In other words, if a DM was assigned an ordinal rank that was higher by a value of 1, it chose the equal allocation (1, 1) 7% more. That is, monkeys with higher assigned ranks were more averse to falling behind.

In a similar vein, recall that the regression of the strength of advantageous inequity concerns on the body weight of the DM had a slope of -0.01 (z score = -1.19, p = 0.235) and was not significant. If we replaced the body weight with the ordinal rank, the slope was again negative at -0.01 (z score = -0.69, p = 0.488) and still not significant. Thus, we did not find that monkeys with higher assigned ranks were more or less averse to getting ahead.

In summary, although we did not have a direct measure of ranks, the assigned ranks exhibited similar effects to the body weight for the results in step 2.

Regarding the two results in step 4, even though the estimated coefficients did not change much, reflecting the high correlation between weight and rank, significance did change for one result.

The first set of regressions addressed whether the predicted strength of disadvantageous inequity concerns explained the degree of impartiality. The coefficient of the predicted strength, now predicted by rank, was 0.15 (z score = 1.40, p = 0.163), and was not significant. Recall that the similar coefficient for weight was 0.18 (z score = 2.89, p = 0.004) and therefore was significant. Hence, the rank predicted result differed from the weight predicted result.

The second set of regressions addressed whether the unpredicted strength no longer explained the degree of impartiality. Here, it was still the case that the unpredicted strength (now by rank) did not explain the degree of impartiality. The coefficient of the residual was -0.02 (z score = -0.17, p = 0.863) and thus was not significant. Recall that the similar coefficient (by weight) was 0.01 (z score = 0.10, p = 0.924) and nonsignificant. Hence, the lack of significance did not change. After removing the rank predicted part, the residual disadvantageous inequity concerns no longer accounted for the impartiality behind the VOI.

We further looked into other factors, including weight difference, assigned ordinal rank difference, gender, gender difference and whether two monkeys were housed in the same cage. None of them explained disadvantageous or advantageous inequity aversion. Specifically, the regression of the strength of disadvantageous inequity concerns on the body weight difference between the DM and the RM had a slope of -0.01 (z score = -0.97, p = 0.330). Similar regressions of the strength of disadvantageous inequity concerns on the assigned ordinal rank difference, gender, gender difference or whether two monkeys were housed in the same cage respectively had a slope of -0.02 (z score = -1.43, p = 0.154), 0.20 (z score = 1.47, p = 0.142), 0.07 (z score = 1.71, p = 0.087), 0.01 (z score = 0.22, p = 0.824). The regression of the strength of advantageous inequity concerns on the body weight difference had a slope of -0.01 (z score = -1.33, p = 0.184). Similar regressions of the strength of advantageous inequity concerns on the assigned ordinal rank difference, gender, gender difference or whether two monkeys were housed in the same cage respectively had a slope of -0.01 (z score = -1.01, p = 0.310), -0.05 (z score = -1.42, p = 0.156), 0.04 (z score = 0.96, p = 0.337), 0.01 (z score = 0.10, p = 0.917). We did not find evidence that other factors explained disadvantageous or advantageous inequity concerns. Therefore, our conclusion stayed the same.

1. Possibility of reciprocation

Some primate experiments took advantage of a large number of subjects so that each DM-RM pair met only once (12). We were unable to do that because we had few monkeys in the lab. Moreover, it required a substantial amount of time to familiarize the monkeys with the apparatus and the design. Hence, each pair met multiple times. In addition to the care we took to avoid the possibility of reciprocation (as described in **Materials and Methods** on the three restrictions when we paired monkeys), below we examined our data to see whether there was evidence of any such influence.

Reciprocation could have occurred in the VOI and Social conditions. If a pair reciprocated each other in the VOI condition, this might be seen if, when the DM chose the equal division frequently, the RM reciprocated by also choosing the equal division frequently when the roles were reversed the next time they met. We thus regressed the proportion a previous RM chose the equal division the next time a pair met on the proportion a DM chose the equal division this time. If reciprocation existed, the coefficient was expected to be significantly positive. The regression coefficient was 0.10 (z score = 0.70, p = 0.484, n = 42), and it was not significant in a two-level linear mixed model. Hence, there was no evidence that monkeys reciprocated in the VOI condition.

In the Social condition, because the DM received the same reward in both allocations, we defined the allocations that gave the RM more as favoring the RM. This translated to the (3, 3) allocation in the Ad condition and the (1, 3) allocation in the Dis condition. If a pair reciprocated each other in the Social condition, we could see this when if the DM favored the RM frequently, the RM reciprocated by also favoring the DM frequently when their roles were reversed. We thus regressed the proportion of the RM’s choices that favored the DM the next time the pair met on the proportion of the DM’s choices that favored the RM this time. If reciprocation existed, the coefficient was expected to be significantly positive. The regression coefficient was 0.01 (z score = 0.06, p = 0.954, n = 42), which was not significant in a two-level linear mixed model. Hence, there was no evidence that monkeys reciprocated in the Social condition.

1. Time effect

We addressed the possibility of time effect in two ways. First, we tested whether choices in any condition changed as time went by. Second, we tested whether choice differences between a condition and its control (VOI vs. Risk, Ad vs. AdC, Dis vs. DisC) were stable. In other words, we tested whether our results were stable.

For the first part, we looked into whether choices differed across blocks. We pooled the data in the Risk and VOI conditions to run a repeated measures ANOVA with condition (Risk or VOI), block, interaction, and DM as factors. The dependent variable was the choice frequency of the equal division. The main effect of the block was not significant (p = 0.3244), and neither was the interaction (p = 0.2326). Hence, choices did not differ across blocks. The block effect did not differ between the Risk and VOI conditions either. Of note, the main effect of the condition (Risk vs. VOI) was significant, consistent with the result we reported in the main text that the equal division was chosen more often in the VOI condition than in the Risk condition.

**ANOVA: Condition (VOI or Risk), Block, Interaction**

| Source | Partial SS | df | MS | F | p-value  Regular | p-value  Box |
| --- | --- | --- | --- | --- | --- | --- |
| Condition | 0.2000 | 1 | 0.2000 | 8.37 | 0.0049 |  |
| Block | 0.0843 | 3 | 0.0281 | 1.18 | 0.3244 | 0.2879 |
| Interaction | 0.1045 | 3 | 0.0348 | 1.46 | 0.2326 | 0.2380 |
| DM | 0.3049 | 3 | 0.1016 | 4.25 | 0.0077 |  |
| Residual | 1.9366 | 81 | 0.0239 |  |  |  |
| Total | 2.5884 | 91 | 0.0284 |  |  |  |

Applying the same ANOVA to the data in the AdC and Ad conditions, we did not observe a block effect (p = 0.0923) or an interaction between block and condition (p = 0.8801).

**ANOVA: Condition (Ad or AdC), Block, Interaction**

| Source | Partial SS | Df | MS | F | p-value  Regular | p-value  Box |
| --- | --- | --- | --- | --- | --- | --- |
| Condition | 0.0725 | 1 | 0.0725 | 3.09 | 0.0866 |  |
| Block | 0.0698 | 1 | 0.0698 | 2.98 | 0.0923 |  |
| Interaction | 0.0005 | 1 | 0.0005 | 0.02 | 0.8801 |  |
| Subject | 0.0626 | 3 | 0.0209 | 0.89 | 0.4547 |  |
| Residual | 0.9145 | 39 | 0.0234 |  |  |  |
| Total | 1.1834 | 45 | 0.0263 |  |  |  |

Similarly, applying the same ANOVA to the data in the DisC and Dis conditions, we did not observe a block effect (p = 1.000) or an interaction between block and condition (p = 1.000).

**ANOVA: Condition (Dis or DisC), Block, Interaction**

| Source | Partial SS | Df | MS | F | p-value  Regular | p-value  Box |
| --- | --- | --- | --- | --- | --- | --- |
| Condition | 0.0115 | 1 | 0.0115 | 0.71 | 0.4058 |  |
| Block | 0.0000 | 1 | 0.0000 | 0.00 | 1.0000 |  |
| Interaction | 0.0000 | 1 | 0.0000 | 0.00 | 1.0000 |  |
| Subject | 0.1653 | 3 | 0.0551 | 3.40 | 0.0272 |  |
| Residual | 0.6338 | 39 | 0.0162 |  |  |  |
| Total | 0.8111 | 45 | 0.0180 |  |  |  |

For the second part, we examined whether our major results held if we split all conditions into two halves. For instance, the VOI condition was split into VOI 1, the first half; and VOI 2, the second half. If time were the reason why the equal division was chosen more often behind the VOI than in the Risk condition, because we ran the Risk condition before the VOI condition, Risk 1 and VOI 2 were most separated in time and hence differences in choices between them could be expected to be large. On the other hand, Risk 2 and VOI 1 were least separated in time. The difference between them, by the same reasoning, could be expected to be small. We intended to see whether the VOI vs. Risk difference was primarily driven by time, so that VOI 2 vs. Risk 1 would be significantly different but VOI 1 vs. Risk 2 would not.

We found that the equal division was chosen significantly more often in two combinations (including VOI 1 vs. Risk 2, least separated in time; and VOI 2 vs. Risk 2, which had an intermediate separation of time). In the remaining two combinations (VOI 1 vs. Risk 1, with an intermediate separation of time; VOI 2 vs. Risk 1, most separated in time), the equal division was still chosen more often in VOI. The p-values were 0.051, 0.057 respectively, right on the margin of significance. Hence, the increased choice of the equal division in VOI was quite stable. It was not driven by a particular combination that was most separated in time, namely VOI 2 vs. Risk 1. This supported that the elapsed time between VOI and Risk did not influence the results. We cautioned that the use of only a quarter of our data in each combination would make estimations noisier and less likely to be significant.

We next examined disadvantageous inequity concerns. Before, we showed that the strength was not significantly different from zero; now, it was still so in all four combinations (the smallest p-value among the four combinations was 0.542). Before, body weight could significantly account for the strength positively; now this was still the case in all four combinations (the largest p-value among the four combinations was 0.026). Hence, the results of disadvantageous inequity concerns were stable.

Regarding advantageous inequity concerns, before, we observed a significant tendency to dislike getting ahead, but body weight could not account for it. Here, we needed to qualify our earlier results. Though body weight still could not account for it (the smallest p-value among the four combinations was 0.455) and the tendency was still to dislike getting ahead, we did not find significance in all combinations. We found one strong significance (p-value smaller than 0.001) and three insignificances (p = 0.067 in one combination and p-value greater than 0.1 in two remaining combinations). Though the average tendency was significantly positive, this did not apply to all combinations. Again, the use of a smaller amount of data made estimations noisier and less likely to be significant.

Overall, the impartiality behind the VOI and disadvantageous inequity concerns were robust even when data were split into halves and all possible combinations were considered. The advantageous inequity concerns were not significant in all combinations and needed to be qualified. Because our main contribution regarded VOI choices and the connection between VOI choices and disadvantageous inequity aversion, the conclusion of our results held up when the time effect was considered.

(7) Non-parametric analyses

We split the sample based on the DM level (cluster) and constructed the bootstrap sample by drawing clusters with replacement. Then we re-estimated the treatment effect based on the bootstrap sample. We repeated this procedure 10000 times and used those estimates to construct the standard error. The degree of impartiality was estimated to be 0.11 (p < 0.001). The strength of advantageous inequity concerns was 0.11 (p < 0.001). The strength of disadvantageous inequity concerns was -0.05 (p = 0.503). Regressing the strength of disadvantageous inequity concerns on the body weight, the coefficient was 0.05 (p = 0.048). Regressing the strength of advantageous inequity concerns on the body weight, the coefficient was -0.01 (p = 0.446). Regressing the degree of impartiality on the strength of disadvantageous inequity concerns, the coefficient was 0.15 (p = 0.001). Regressing the degree of impartiality on the strength of advantageous inequity concerns, the coefficient was -0.15 (p = 0.100).

Overall, our results held up. We still found evidence of impartiality. We still found that heavier monkeys showed a stronger aversion to falling behind. Finally, we still found a positive correlation between the strength of disadvantageous inequity concerns and the degree of impartiality behind the VOI.

**Captions for Movies**

**Movie S1**

VOI: The top panel of the movie shows a view toward the monkeys, and the bottom panel shows a view toward the food trays. When facing the monkeys, the DM (Little Babe) is on the right and the RM (Coffee) on the left. The upper tray is baited with (3, 1) where 3 is in the left dish and the lower with (2, 2). The DM pulls the upper bar. The upper tray is then spun one and a half loops.

**Movie S2**

Risk: The upper tray is baited with (2, 2) and the lower with (1, 3) where 1 is in the left dish. The DM (Venus) pulls the lower bar. The lower tray is then spun one and a half loops.
